# A Case of Epiphysial Fracture of Humerus, with Dislocation

**Published:** 1878-07

**Authors:** Frederick Peterson


ART. II.—*A Case of Epiphysial Fracture of Humerus, with Dislocation.* Reported by FREDERICK PETERSON. Sisters of Charity Hospital—Surgical service of Dr. J. CRONYN.

J. M., æt. 19, a sailor; April 27, 1878, came into the Hospital, having a swelling of two inches in diameter under the inner fibres of the Deltoid, on the antero-interior aspect of the right upper extremity. On interrogation in regard to history, it was discovered that some three years before, in a sack race, he had fallen and injured his arm in a manner which led to its being described as a luxation of the shoulder-joint. From the effects of this he entirely recovered. After a time the swelling appearing in the locality mentioned, he obtained free exercise of the joint. A few weeks previous to entering the Hospital he had the misfortune to receive a second shock on the shoulder, rendering him unfit for labor; upon which event he decided to have the tumor removed. The eminence prior to the last accident occupied a higher situation. It was found on examination to be intensely hard. The length of time elapsed since its first appearance, the epiphysial age of the patient, the immediate advent of the swelling, the manner of obtaining the injury, and all subsequent developments, led to the suspicion that it was the superior epiphysis of humerus. This being diagnosed, the removal was decided upon with the anticipation of speedy recovery from the operation.

April 29th the excision was undertaken, patient being under the influence of chloroform. An incision nearly three inches in length was made above the tumor, and proceeding through the inner fibres of the Deltoid and fascia of the Pectoralis major, the epiphysis, as it proved to be, was extracted on severing an elongated spicula of bone, part of the lesser tuberosity, with a contracted portion of synovial membrane which had burst through the capsule of the joint. That this was the synovial membrane was proven by the efflux of a quantity of limpid, unctious synovial fluid. The wound was quickly brought together with a pin-suture, a compress placed on inner aspect, and arm bandaged to the body; the object of this procedure being to procure the rapid healing of the ruptured membrane, and prevent the usual consequences of an opening into the the joint. Though the wound closed by first intention, inflammation took place, and in a day or two it was freely opened to allow the escape of the purulent matter which had formed. A slight erysipelatous blush was noticed. May 4, unfortunately, he was found in a high rheumatic fever, a sequence of prior exposure, tongue thickly furred, pulse 96, anxious expression of countenance, and all joints more or less affected. This circumstance retarded recovery to a great degree, but under treatment of salicylate of soda in twenty-grain doses every four hours, an immediate arrest was made to its farther progress. On account of continuance of arthritic pains, iodide of potash was substituted in a few days with agreeable results. From this time forward his convalescence was rapid. Now, May 19, he is wholly well. The rheumatism has left him entirely. He has regained the perfect use of his joint, its movement in any direction not being impeded, and equal in all respects to the opposite articulation.

The case, as detailed above, is interesting and unique for this reason, that it is, as far as we know, the only one of its character in that situation on record, though fractures of the anatomical neck, with impaction and osseous, cartilaginous or ligamentous adhesion, are met with occasionally.* At the time of separation of epiphysis, the capsule was ruptured, but the piece of bone carried the synovial membrane forward with it. The epiphysis had, during the three years, by muscular action and as a result of the second injury, moved lower down. A new head to the humerus has of course been formed by bony deposition.

:o:

Dr. Orlaw, of St. Petersburg, reports ten successful cases of injection of the tincture of iodine into the knee-joint for chronic inflammation. The strength used was one drachm of tincture to three of water. Effusions in the cavity of the joint were first evacuated. The inflammation was rapidly ameliorated, and there was no relapse.—*Boston Medical Journal.*

*Prof. Miner had a similar case occurring in the head of the fermur, in which there was dislocation and fracture of neck of fermur, the head being removed immediately after the injury.

